# Volumetric BOLD fMRI simulation: from neurovascular coupling to multivoxel imaging

**DOI:** 10.1186/1471-2342-12-8

**Published:** 2012-04-23

**Authors:** Zikuan Chen, Vince Calhoun

**Affiliations:** 1 The Mind Research Network and LBERI, Albuquerque 87106, NM, USA; 2 Department of Electrical and Computer Engineering, Albuquerque 87131, NM, USA

**Keywords:** Bold fMRI, Neurovascular coupling, Neuroactive blob (NAB), Blood magnetism, Intravoxel dephasing, Voxelization, Magnetic fieldmap, Spatial correlation

## Abstract

**Background:**

The blood oxygenation-level dependent (BOLD) functional magnetic resonance imaging (fMRI) modality has been numerically simulated by calculating single voxel signals. However, the observation on single voxel signals cannot provide information regarding the spatial distribution of the signals. Specifically, a single BOLD voxel signal simulation cannot answer the fundamental question: is the magnetic resonance (MR) image a replica of its underling magnetic susceptibility source? In this paper, we address this problem by proposing a multivoxel volumetric BOLD fMRI simulation model and a susceptibility expression formula for linear neurovascular coupling process, that allow us to examine the BOLD fMRI procedure from neurovascular coupling to MR image formation.

**Methods:**

Since MRI technology only senses the magnetism property, we represent a linear neurovascular-coupled BOLD state by a magnetic susceptibility expression formula, which accounts for the parameters of cortical vasculature, intravascular blood oxygenation level, and local neuroactivity. Upon the susceptibility expression of a BOLD state, we carry out volumetric BOLD fMRI simulation by calculating the fieldmap (established by susceptibility magnetization) and the complex multivoxel MR image (by intravoxel dephasing). Given the predefined susceptibility source and the calculated complex MR image, we compare the MR magnitude (phase, respectively) image with the predefined susceptibility source (the calculated fieldmap) by spatial correlation.

**Results:**

The spatial correlation between the MR magnitude image and the magnetic susceptibility source is about 0.90 for the settings of T_E _= 30 ms, B_0 _= 3 T, voxel size = 100 micron, vessel radius = 3 micron, and blood volume fraction = 2%. Using these parameters value, the spatial correlation between the MR phase image and the susceptibility-induced fieldmap is close to 1.00.

**Conclusion:**

Our simulation results show that the MR magnitude image is not an exact replica of the magnetic susceptibility source (spatial correlation ≈ 0.90), and that the MR phase image conforms closely with the susceptibility-induced fieldmap (spatial correlation ≈ 1.00).

## Background

Blood oxygenation-level dependent (BOLD) functional magnetic resonance imaging (fMRI) has been widely accepted for brain functional mapping and neuroimaging [1-6]. The imaging principle of BOLD fMRI is that: a neuroactivity incurs cerebral vascular blood magnetism perturbation that can be detected by magnetic resonance imaging (MRI). In neurophysiology, the BOLD fMRI can be described by a neurovascular coupling model as follows [7-11]: a neuronal activity incurs a vascular response in terms of changes in cerebral blood flow (CBF), cerebral blood volume (CBV), and cerebral metabolic rate of oxygen (CMRO_2_). The neuroactivity-induced biomagnetic susceptibility perturbation can be detected by T2*-weighted MRI (T2*MRI) [12]. An overall BOLD fMRI model can be decomposed into a cascade of two modules [13]. One is a neurophysiology module that addresses the vascular response to a neuroactivity in context of neurovascular coupling [7-11]; The neurovascular coupling process produces an intravascular blood biomagnetism perturbation that is detectable by T2*MRI [12]. Another is a MRI technology module dedicated to imaging the susceptibility-expressed neurovascular coupling states. Despite its wide acceptance for neuroimaging and brain mapping, the BOLD fMRI mechanism is not fully understood [5,14,15]. Specifically, the complicated neurovascular-coupled BOLD process is not fully understood, and the imaging performance of T2*MRI detection on a BOLD state has never been quantitatively examined in a multi-voxel simulation.

In past decades, there have been many published reports on numerical simulations of BOLD mechanism [1,6,16-18]. To our knowledge, previous BOLD simulations were carried out for investigating single voxel signals and did not address multiple voxels. This may be partially due to the computational burden and the implementation difficulty in manipulating a large 3D image matrix (e.g. 2048 × 2048 × 2048 in our simulation). In practice, a BOLD fMRI experiment produces a 4D dataset that consists of a time series of 3D image matrices (typically in size of 64 × 64 × 32 voxels) captured by T2*MRI at a discrete time point (snapshot). A multivoxel image offers many spatial features, such as "contrast and texture", "geometry: edge/shape/pattern", "topography", "topology", and so on. Furthermore, given an input source and its corresponding output image, we can assess its overall imaging performance in terms of point spread function, spatial invariance/variance, and linearity/nonlinearity of a digital imaging system.

Since T2*MRI is designed to sense an inhomogeneous fieldmap that is established via magnetization of an inhomogeneous susceptibility distribution, the underlying source of fMRI is the susceptibility-expressed distribution of a neurovascular coupling state. Therefore, for numerical BOLD fMRI simulation, we need numerically characterize the neurovascular coupling process in terms of biomagnetism susceptibility perturbation for the purpose of T2*MRI detectibility. Given a susceptibility map (representing a snapshot of dynamic BOLD susceptibility perturbation), we can carry out T2*MRI simulation in a way similar to BOLD voxel signal simulations [19-21]. Intuitively, a multivoxel BOLD fMRI simulation may be implemented by spatially arranging individual voxel signal values. In the implementation, nonetheless, we need account for the electromagnetic interaction among the voxels (due to the nonlocal effect of vascular blood magnetization [22]), especially for the magnetic influence from vasculatures in surrounding voxels that may influence the BOLD voxel signal by 10% [23].

In this paper, we propose a volumetric BOLD fMRI model that deals with a cortical field of view (FOV), as a whole matrix (without dividing into submatrices), in which the magnetic influence among the intra-FOV vasculatures are accounted for during the fieldmap calculation using a Fourier technique [16,23-25]. After calculation of the fieldmap over the FOV, we proceed to calculate the output image of T2*MRI based on intravoxel dephasing. Upon the forward BOLD fMRI simulation, we obtain a complex-valued image (consisting of a pair of magnitude and phase parts) at each snapshot time. By comparing the MR magnitude image with the susceptibility source in a measure of spatial correlation, thereby evaluating how well the susceptibility source can be reproduced in the MR magnitude image. Meanwhile, we may compare the MR phase image with the fieldmap, thereby justifying the practice of fieldmap measurement by MR phase imaging. It is noted that the fieldmap is spatially different from the susceptibility source by a 3D convolution [13,22,26], therefore, we do not expect a morphological match between the susceptibility map and either the fieldmap or the phasemap.

The neurovascular coupling process is a complicated neurophysiological process. Experiments have shown that the BOLD activity in response to a neuroactivity may be described as a linear response model [7,8,27-32]. For the implementation of numerical simulation, we propose a linear neurovascular coupling formula to account for the biomagnetic susceptibility contributions from various neurovascular coupling aspects, such as neuroactivity (NAB: neuroactivity blob), vasculature (CBV: cerebral blood volume), blood physiology (CBF: cerebral blood flow), and blood metabolism (CMRO_2_:cerebral metabolism rate of oxygen).

A BOLD fMRI study produces a 4D MR dataset, of which each 3D volume at a time point is interpreted as a snapshot of a dynamic BOLD state. The 4D BOLD fMRI data acquisition can be numerically simulated by a train of 3D T2*MRI snapshots provided the dynamic susceptibility perturbation is numerically specified for each snapshot time. In this report, we will only focus on the snapshot capture of a susceptibility-expressed BOLD physiological state at a specific time point. Our approach is general and we can implement a dynamic 4D BOLD fMRI simulation by repeating the snapshot imaging at a series of time points at more computation cost.

## Methods

**Figure 1:**
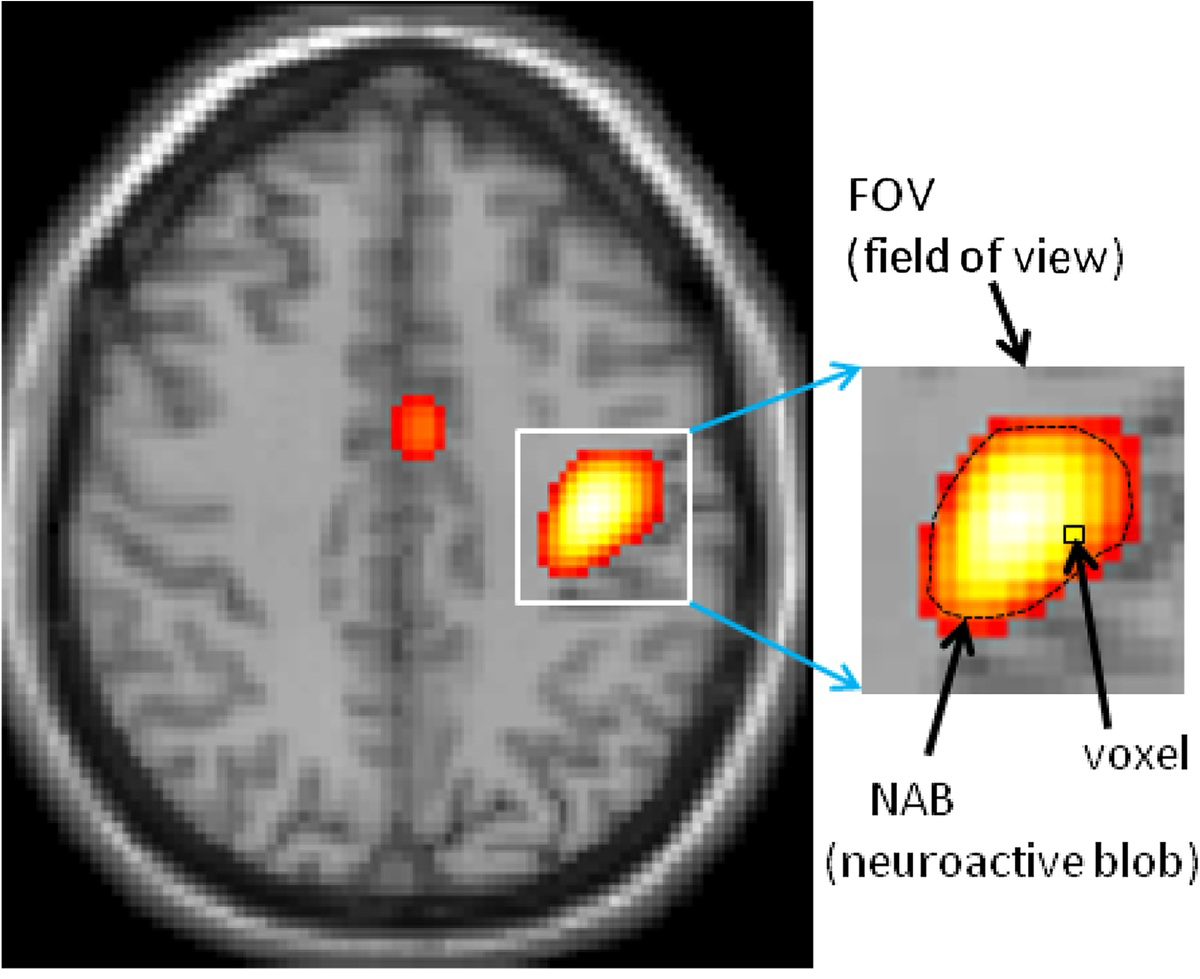
**Illustration of a local neuroactive blob (NAB) observed at cerebral cortex by fMRI experiment**. Conventionally, a NAB is inferred from an fMRI data and is considered as a local neuronal activity. However this convention has not been numerically verified from fMRI physics. This paper proposes a volumetric BOLD fMRI computation model to quantitatively examine the multivoxel fMRI formation of a magnetic susceptibility expressed BOLD state under a linear NAB-modulated neurovascular coupling assumption. Simulations are rendered over a 3D cortical FOV that contains a NAB with adequate margins in an array of vasculature-laden voxels. (The image shown in Figure 1 was taken with the written consent of the subject for a separate study in our group. The image acquisition protocol was approved by the Mind Research Network review board).

**Figure 2:**
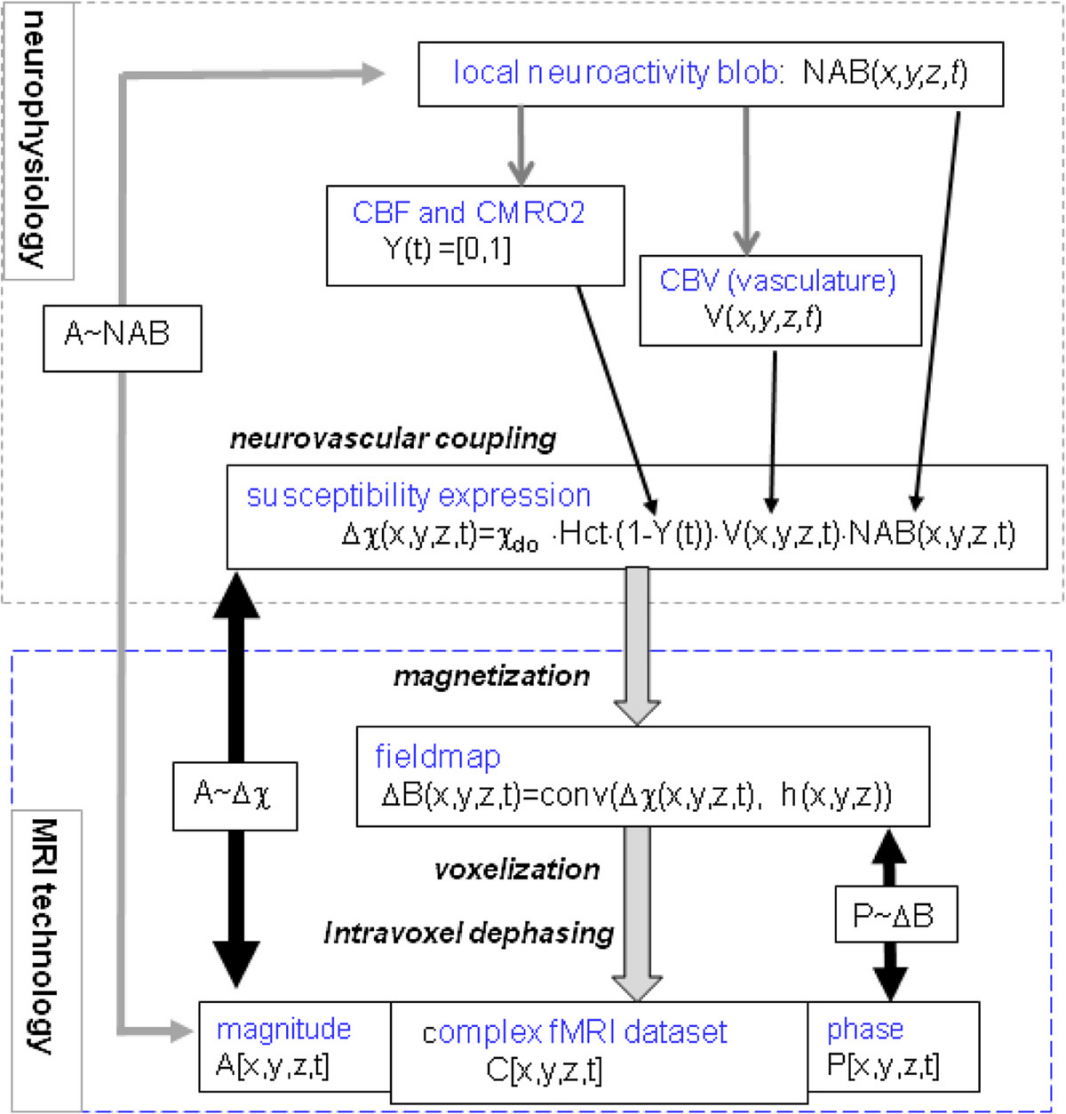
**Overall diagram of the computational model for neurovascular coupling and BOLD fMRI**. It is decomposed into a neurophysiology module (upper dashed box) and a MRI technology module (down dashed box). The linear neurovascular coupling model accounts for the local neuroactivity, blood oxygenation, and cortical vasculature by a spatiotemporal modulation. The complex fMRI dataset is resulted from susceptibility magnetization and intravoxel dephasing (T2* imaging). The complex fMRI dataset can be used for backward mappings as diagrammed by double-directed arrows. It is noted the conventional neuroimaging is diagrammed by an overall backward mapping as diagrammed by a grey double-directed arrow of A ~ NAB on the left-hand side. In this paper, we focus on the mapping from the MR magnitude image to the susceptibility source (as diagrammed by the black double-directed arrow of A~Δχ), and that from the MR phase image to the fieldmap (P~ΔB).

We motivate our approach by looking at a typical neuroimage as shown in Figure 1, which can be modeled as a population of local neuroactivity blobs (NAB). A spatially localized NAB is inferred from an fMRI dataset and is interpreted as (or related to) the underlying neuronal activity (neuronal origin). Because this inference is based on MR magnitude images, it is important to evaluate how well a MR magnitude image can represent a snapshot of the neuronal origin. Since the T2*MRI detection is a typical noninvasive 3D imaging modality, it is possible (virtually necessary) to quantitatively assess the neuronal origin inference from MR images by following the medical imaging principle: predefining an input source, predicting its output image by numerical simulation (or phantom experimental verification when applicable), and then comparing the obtained output image with the predefined input source. Accordingly, we address the NAB inference issue in Figure 1 by numerically simulating the neurovascular coupling process and the BOLD fMRI detection technology by the following steps: 1) delineating a cortical FOV that is ample enough to enclose a NAB, as demonstrated in Figure 1; 2) defining a susceptibility-expressed BOLD state under the NAB-modulated neurovascular coupling; and 3) carrying out the multivoxel BOLD fMRI simulation for a predefined susceptibility source. Upon the completion of BOLD fMRI simulation, we compare the MR magnitude image with the predefined susceptibility source in a measure of spatial correlation. A key benefit of our approach is we produce a full 3D image in form of a multivoxel matrix, in contrast to the many previous studies which focused only on one or a few voxels.

The overall diagram for the computational model of neurovascular coupling and BOLD fMRI is shown in Figure 2, which consists of two cascaded modules: 1) the "neurophysiology" module for the neurovascular coupling process from a neuronal origin to its vascular response, which should be phenotypically expressed in terms of biomagnetic susceptibility distribution for the purpose of MRI detection, and 2) the "MRI technology" module for imaging a susceptibility-expressed BOLD state by T2*MRI. According to this two-module BOLD fMRI model, the neuronal origin is a NAB-expressed neuroactivity (in response to an external stimulus, not shown), and the vascular response is a result of neurovascular coupling (numerically expressed in form of Δχ), which in turn serves as the vascular origin of T2*MRI detection. Conventionally, the goal of brain mapping and neuroimaging is to depict the neuroactivity origin from MR magnitude images, as diagrammed by a grey double-directed arrow for the backward mapping as designated by "A ~ NAB". Based on the two-module model in Figure 2, the overall backward mapping of "A ~ NAB" can be decomposed into two steps: the first step is from the MR magnitude image to the vascular origin, as denoted by a black double-directed arrow and designated by "A~Δχ", the second step is from the vascular origin to the neuronal origin (not shown in Figure 2). In this paper, we will look into the first step backward mapping of "A~Δχ" by numerical T2*MRI simulation, and simplify the second step backward mapping by a linear neurovascular coupling model. Meanwhile, we also show another backward mapping: from a MR phase image to fieldmap as diagrammed by a black double-directed arrow as designated by "P~ΔB" in Figure 2.

### Neurovascular coupling formulation

It is known in neurobiology and neurophysiology that a neuroactivity is accompanied by a complicated process of cellular, metabolic, and vascular processes. For simplification of computational implementation, we express the spatial distribution for the functional parcellation of a neuroactivity by a 3D Gaussian-shaped NAB embedded in a cortical FOV (with size D_0 _× D_0 _× D_0_) by

(1) $$NAB\left(x,y,z\right)=c\text{exp}\left(-\frac{{\left(x-x_{0}\right)}^{2}}{\sigma_{x}^{2}}-\frac{{\left(y-y_{0}\right)}^{2}}{\sigma_{y}^{2}}-\frac{{\left(z-z_{0}\right)}^{2}}{\sigma_{z}^{2}}\right)\text{,}\;|x-x_{0}|\leq \frac{D_{0}}{2},\;|y-y_{0}|\leq \frac{D_{0}}{2},\;|z-z_{0}|\leq \frac{D_{0}}{2}$$

where (*σ_x_, σ_y_, σ_z_*)delineates an ellipsoidal profile (*σ_x _*= *σ_y _*= *σ_z_*) for sphere, *σ*_x _= *σ*_y _<<*σ*_z _for long ellipsoid or cylinder, *σ*_x _≠ *σ*_y _≠ *σ*_z _for general ellipsoid), (x_0_, y_0_, z_0_) denotes the location of the NAB in the FOV, and c = max(NAB) represents the maximum activity at the NAB center (the activity strength is scaled by 0 < c < 1). We should mention that a non-spherical blob like the one in Figure 1 can be formulated by a combination of several regular Gaussian-shaped NAB primitives that are different in (x_0_, y_0_, z_0_) and (*σ*_x_, *σ*_y_, *σ*_z_). The graded local neuroactivity strength over the FOV is reflected in the spatially distributed multivalues in the range of [0, c] (maximum activity strength at the NAB center, moderate strength at the NAB boundary, and zero strength outside the NAB).

**Figure 3:**
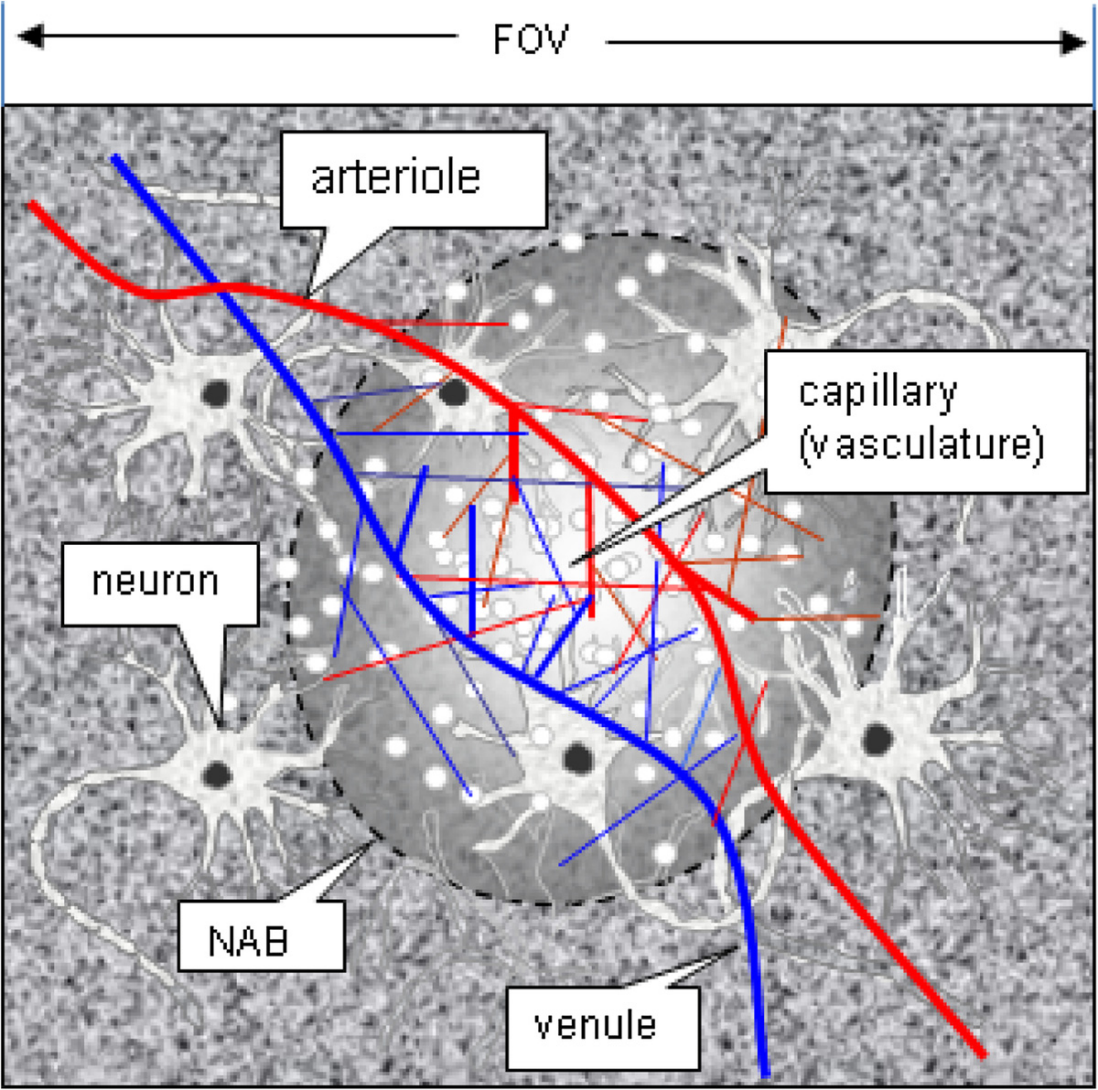
**A 2D illustration of a localized neurovascular coupling model**. The local neuroactivity is represented by a ball-shaped neuroactive blob (NAB), which confines the locality and the graded strength in a cortical FOV. The red and blue lines illustrate the oxygen-rich arterioles and oxygen-poor venules. Due to spatial weighting by NAB, the cortical regions outside the NAB has no contribution to the BOLD state. (In numerical BOLD fMRI simulation, the neuron-glial clusters are considered as the extravascular parenchyma, thus being omitted).

In response to the neuroactivity in a NAB, the neurovascular unit regulates the cerebral microcirculation by vasodilation (increase in CBV) and a burst of blood inflow (increase in CBF). The intravascular blood oxygenation level is subject to change due to neuromodulation in the NAB, primarily occurring in the capillaries and venules. Figure 3 illustrates a neurovascular coupled blood oxygen delivery in capillary bed confined in a NAB. It is shown that the BOLD activity is spatially localized in a cortex region (NAB confinement) where the central region undergoes the maximal activity, and there is no neuronal activity outside NAB. The blood biomagnetic susceptibility perturbation is determined by the spatial modulation between the NAB distribution and the vasculature geometry. The intravascular blood volume (CBV) is determined by the vasculature geometry at a snapshot pose, and the dynamic intravascular blood flow (CBF) is determined by the intracranial blood pressure, vasculature geometry and blood fluid mechanics. All in all, for numerical simulation purpose, all these neurovascular parameters should be numerically expressed in terms of magnetic susceptibility responses (detectable by MRI technology).

At a snapshot time, the cortical vasculature geometry determines the intravascular blood volume, that only takes up a fractional space of the cortical FOV, as described by blood volume fraction (*bfrac*). For human cerebral cortex, *bfrac *≈ 2% [33]. For numerical simulation of 3D vasculature-laden FOV, we express the vasculature configuration by a binary volume that is randomly generated under the control of *bfrac*. That is,

(2) $$\begin{array}{c} V\left(x,y,z,t\right)=\left\{\begin{array}{cc} 1, & \left(x,y,z\right)\in \text{vessel}\\ 0, & \text{otherwise} \end{array}\right),\text{ with }dom\text{(}V\text{)}=FOV\\ \text{and }bfrac\left(t\right)=\frac{\underset{\left(x,y,z\right)}{\sum }V\left(x,y,z,t\right)}{\left|V\right|} \end{array}$$

where *dom*(V) denotes the space domain of the selected cortical vasculature, and *bfrac*(*t*) the dynamic blood volume fraction, and |V| the volume of FOV. It is noted that the time parameter t is explicitly used to indicate the variation in vasculature geometry. We should point out that we can use *bfrac*(t) to numerically characterize the blood physiological parameters: CBV and CBF. Specifically, a vasodilation associated with CBV manifests as a slight increase in *bfrac*(t), that is *bfrac*(t+Δt) >*bfrac*(t) for Δt > 0; and an accelerated blood flow can be equivalent to an increase in *bfrac*(t) as well. Since *bfrac *plays a control parameter during random vasculature generation (in Eq. (2)), its effect on intravascular blood physiology is exerted through the vasculature configuration geometry.

It is also known that only the red blood cells in blood stream convey oxygen that contribute to intravascular blood susceptibility perturbation. The total volume of red blood cells in normal blood is about 40%, as described in terms of hematocrit (Hct≈0.4). Blood physiology also shows that a red blood cell can carry up to 4 oxygen molecules (via attachment to 4 heme groups in a hemoglobin). Due to the oxygen detachment during microcirculation, the deoxygenated blood reveals more paramagnetism than the oxygenated blood, that is *χ*_deoxy _>*χ*_oxy_. Let Y(t) represent the dynamic blood oxygenation level, then (1-Y(t)) represents the dynamic deoxygenation level, that is a parameter to reflect the cerebral metabolism of oxygen (CMRO_2_). It is noted that Y(t) = [0,1], with Y = 1 for the fully oxygenated blood in artery and Y = 0 for the fully deoxygenated blood in vein.

Based on the neurovascular-coupled blood biomagnetic perturbation mechanism, we propose a biomagnetic susceptibility expression formula for a linear neurovascular coupling model by

(3) $$\begin{array}{c} \chi_{total}\left(x,y,z,t\right)=\left[\chi_{deoxy}\cdot \left(1-Y\left(t\right)\right)+\chi_{oxy}\cdot Y\left(t\right)\right]\cdot Hct\cdot NAB\left(x,y,z\right)\cdot V\left(x,y,z,t\right)+\chi_{tissue}\left(x,y,z\right)\\ \chi_{base}\left(x,y,z\right)\equiv \chi_{oxy}\cdot Hct\cdot NAB\left(x,y,z\right)\cdot V\left(x,y,z\right)+\chi_{tissue}\left(x,y,z\right)\\ \Delta \chi \left(x,y,z,t\right)\equiv \chi_{total}\left(x,y,z,t\right)-\chi_{base}\left(x,y,z\right) \end{array}$$

where *χ*_do _= *χ*_deoxy_-*χ*_oxy _= 0.27 × 4*π*ppm (SI unit) represents the magnetic susceptibility change between deoxyhemoglobin and oxyhemoglobin, which has been used for BOLD signal simulation [17,23]. The total susceptibility χ_total _includes contributions from both intravascular blood and extravascular tissue parenchyma. The susceptibility distribution of a selected baseline state is denoted by χ_base_. In reference to χ_base_, we can characterize a BOLD susceptibility perturbation state by Δχ in Eq. (3). Suppose the BOLD fMRI system is a linear digital imaging system, then the behavior of MR image change in reference to its baseline state can represent the intravascular susceptibility perturbation Δ*χ*(x, y, z, t). In other words, the linear BOLD fMRI model allows us to infer the intravascular BOLD susceptibility perturbation by observing the corresponding MR image change (in reference to their respective baselines). From Eq. (3), we can express the NAB-modulated BOLD susceptibility perturbation state by

(4) $$\Delta \chi \left(x,y,z,t\right)=\chi_{do}\cdot \left(1-Y\left(t\right)\right)\cdot NAB\left(x,y,z\right)\cdot V\left(x,y,z,t\right)\cdot Hct$$

This is a computational model for linear neurovascular coupling, which provides a mathematical formula for numerically expressing the NAB-modulated BOLD response process in a vasculature-filled FOV. Specifically, CMRO_2 _is accounted for by Y(t), CBF and CBV by *bfrac*(t) that is implicitly embodied in V(x, y, z, t) via vasculature configuration (during vasculature geometry generation under the condition of *bfrac*(t) in Eq. (2)), and the local neuroactivity by NAB(x, y, z) which confines the neuroactivity extent and defines a graded neuroactivity strength. Usually, the blood magnetism parameter χ_do _and the blood physiology parameter Hct assume for normal blood, which are experimentally determined constants (see Table 1).

**Table 1:** Parameters and settings for numerical simulations

Parameters	Settings	Remarks
B_0_	3 Tesla	Main static magnetic field

Hct	0.4 (dimensionless ratio)	Blood hematocrit

Y	0.6 (dimensionless ratio)	Oxygenation level = [0,1]

χ_do_	0.27 × 4π ppm (SI metrics)	= χ_dexoy _- χ_oxy_

NAB	NAB(x, y, z) Gaussian ellipsoid	Neuroactive blob (Eq(1))

V(x, y, z)	Binary volume (radii = [2,10] μm)	Cortical vasculature (Eq(2))

FOV: D_0 _× D_0 _× D_0_	2 × 2 × 2 mm^3^	Cortical field of view

FOV support matrix	2048 × 2048 × 2048	Digital geometry (1 μm grid)

voxel: d_0_× d_0_× d_0_	32 × 32 × 32, 64 × 64 × 64, 128 × 128 × 128	Three voxel sizes (1 μm grid)

C[x_n_, y_n_, z_n_;T_E_]	64 × 64 × 64, 32 × 32 × 32, 16 × 16 × 16	Complex multivoxel image

A[x_n_, y_n_, z_n_;T_E_]	Assuming non-negative	MR magnitude image(Eq(9))

P[x_n_, y_n_, z_n_;T_E_]	Assuming positive/zero/negative	MR phase image (Eq(9))

corrA	In a range of [0, 1]	Magnitude mapping (Eq(10)

corrP	In a range of [0, 1]	Phase mapping (Eq(11))

T_E_	In a range of [0, 60] ms	Gradient echo time

For the neurovascular coupling formula in Eq. (4), we need to point out following aspects:

1. It is a linear neurovascular coupling formula that accounts for different neurovascular parameters by a spatiotemporal modulation. For simplicity, we do not consider the hemodynamic time lag, spatial displacement, or spatial response spread in this work, though our model will support it.

2. The BOLD susceptibility perturbation is due to the temporal modulation by the blood deoxygenation level (1-Y(t)), which is an embodiment of CMRO_2_. The interplay among CBF, CBV and vasculature are numerically characterized by a single parameter *bfrac*(t) that reflects the blood dynamic through vasculature configuration (V(x, y, z, t)).

3. Only intravascular blood deoxyhemoglobin contributes to the BOLD susceptibility perturbation; no contribution from extravascular tissue (due to V(x, y, z) = 0 for extravascular region), nor from oxyhemoglobin (due to 1-Y = 0 for Y = 1), nor from neuronal inactive regions (due to NAB = 0).

4. The volumetric computational model can be considered as a generalization of the single voxel neurovascular coupling model (Δ*χ *= *χ*_do_⋅Hct⋅(1-Y)) that has been accepted for single voxel BOLD signal simulation [1,3,17,23].

**Figure 4:**
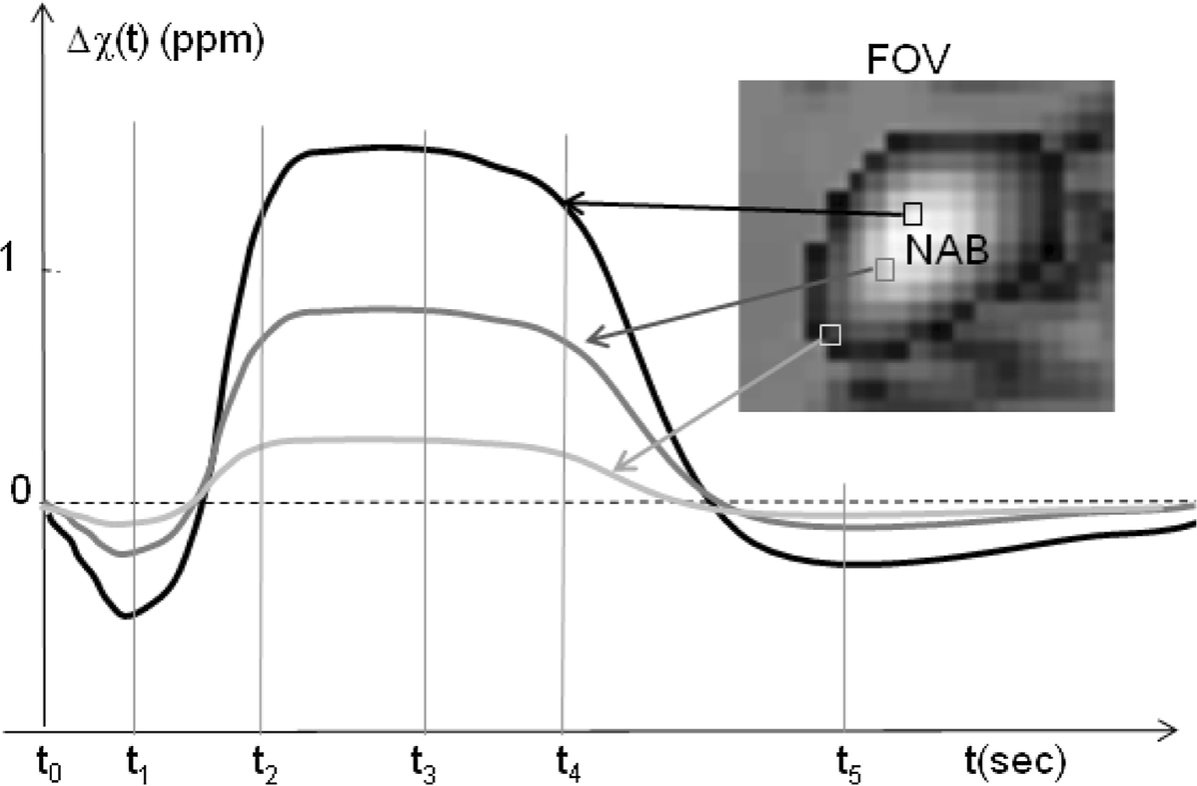
**Illustration of dynamic magnetic susceptibility perturbation in a NAB-weighted cortical region**. It is shown that the voxel at the central NAB region produces the maximum susceptibility perturbation (in comparison with the voxels outside the central NAB), and the dynamic susceptibility perturbation may assume a pres-stimulus transient initial dip (at t_1_) and small post-stimulus undershoot (at t_5_) in addition to the prevailing response mode (between t_2 _and t_4_). For dynamic BOLD fMRI simulation, the neurovascular coupling process should be numerically characterized by a dynamic magnetic susceptibility perturbation in a cortical region. It is noted that the voxel susceptibility timecourses are not necessarily mathematically tractable for numerical representations.

Overall, a multivoxel BOLD fMRI simulation requires a predefined magnetic susceptibility distribution as the input source of T2*MRI. The susceptibility expression for a neurovascular coupling process plays a bridge between the neuroscience and MRI technology. Although the neurovascular coupling process is not fully understood so far, we propose a linear spatiotemporal modulation model (in Eq. (4)) that allows us to look into the effects of CBF, CBV, CMRO_2_, and NAB on the BOLD susceptibility perturbation (which is detected by T2*MRI). In Figure 4, we illustrate a dynamic susceptibility perturbation by susceptibility timecourses of 3 voxels in a NAB, which shows that the susceptibility perturbation strength is spatially weighted by the NAB (maximum at the center and reduced toward boundary). According to Eq. (4), the numerical characterization of neurovascular-coupled 4D dynamic susceptibility perturbation Δχ(x, y, z, t) involves the following conditions: defining a local neuroactivity (a numerical NAB that is not necessarily in an analytic formula), generating a cortical vasculature (V(x, y, z, t)) under the control of *bfrac*(t), and assigning a value to the blood oxygenation level Y(t) (= [0, 1]). It is mentioned that if the digitization of Δχ(x, y, z, t) are experimentally or empirically available for a train of discrete time points (such as at the ticks t_0 _through t_5 _in Figure 4), it is not necessary to seek the analytic formula in Eq. (4). In the following, we will implement the numerical T2*MRI simulation for acquiring a complex MR image from a given susceptibility distribution at a snapshot time, denoted by Δχ(x, y, z), thus omitting the time variable t.

### Forward BOLD fMRI simulation

#### From magnetism perturbation to fieldmap establishment

Given a snapshot of neurovascular-coupled BOLD state, Δχ(x, y, z), we can calculate its magnetization field distribution (resulting from the blood magnetization in a main field B_0_), called the fieldmap henceforth, by [24,25,34]

(5) $$\begin{array}{ccc} \Delta B\left(x,y,z\right) & =IFT\left\{\left(\frac{k_{z}^{2}}{k_{x}^{2}+k_{y}^{2}+k_{z}^{2}}-\frac{1}{3}\right)\cdot FT\left[\Delta \chi \left(x,y,z\right)\right]\right\}B_{0} & \\ & =B_{0}\cdot conv(\Delta \chi \text{(}x,y,z),h\left(x,y,z\right))\\ \text{with }h\left(x,y,z\right) & =\frac{3z^{2}-\left(x^{2}+y^{2}+z^{2}\right)}{4\pi {\left(x^{2}+y^{2}+z^{2}\right)}^{5/2}}\;(\text{point dipole field kernel}) \end{array}$$

where *FT *and *IFT *stand for Fourier transform and inverse Fourier transform respectively, (*k*_x_, *k*_y_, *k*_z_) coordinates in the Fourier domain, conv the convolution, and *h(x, y, z) *the kernel of magnetic point dipole field.

#### Multivoxel partition of FOV

**Figure 5:**
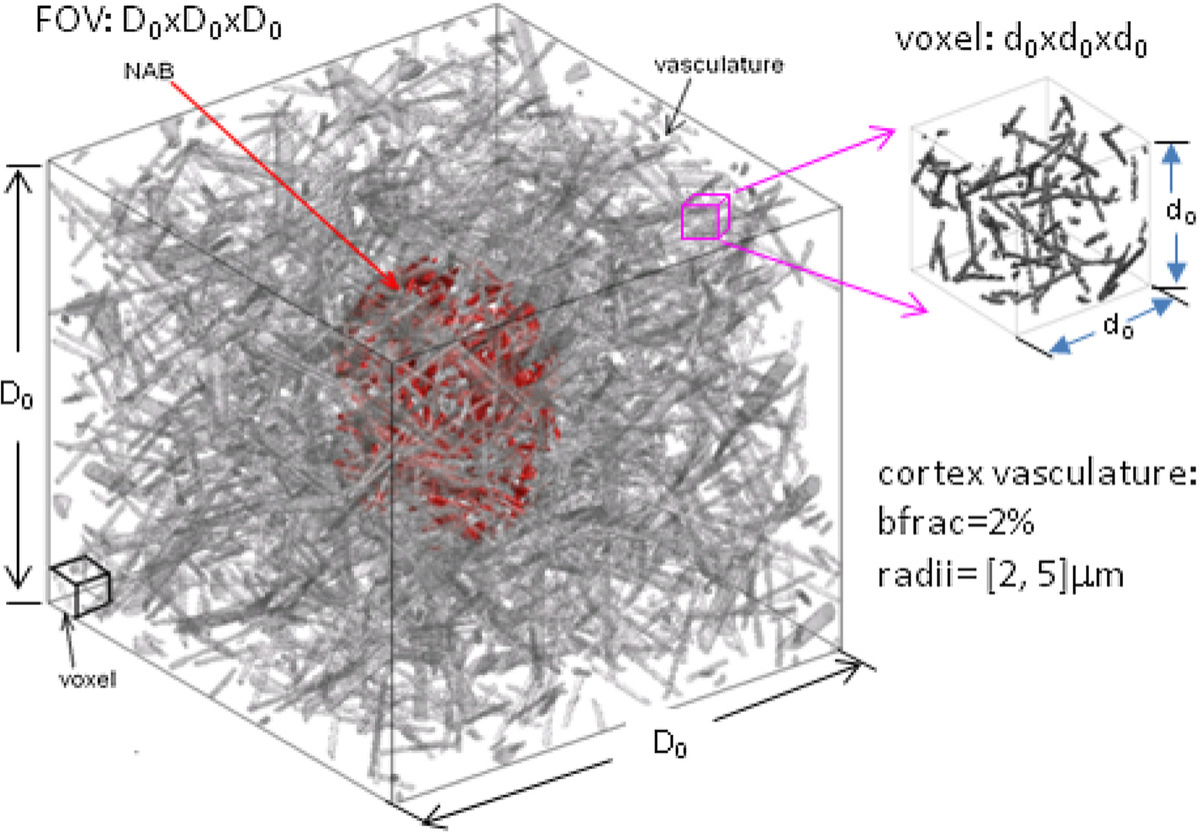
**A typical geometry of cortical FOV that is filled with vasculature and encloses a local neuroactivity blob (NAB in red)**. The fMRI-detectable neurovascular coupling state is expressed as a NAB-weighted intravascular blood magnetic susceptibility perturbation distribution. The vasculature-laden FOV in size of D_0_xD_0_xD_0 _is voxelized by a voxel size of d_0_xd_0_xd_0_, thus producing a reduced matrix in size of [D_0_/d_0_, D_0_/d_0_, D_0_/d_0_]. The vasculature is randomly generated under a control of blood volume fraction (*bfrac*(t)), that is a parameter we use to numerically characterize CBF and CBV.

We simulate the cortical FOV by filling it with vasculature. Figure 5 shows a 3D FOV of size D_0 _× D_0 _× D_0_, which is filled with random vascular networks (the cortical vasculature generation technique has been reported previously [20,23]). The FOV is partitioned into voxels (voxel size = d_0 _× d_0 _× d_0_), thereby we can represent the FOV in a small array of voxels, in which each voxel can be assigned a value by intravoxel average. The process of spatial partition into voxels and intravoxel average is called voxelization. For the vasculature V(x, y, z) in the FOV, the voxelization is expressed by

(6) $$\begin{array}{c} V\left[x_{n},y_{n},z_{n}\right]=V\left(x,y,z\right)*rect\left(\frac{x-x_{n}}{d_{0}},\frac{y-y_{n}}{d_{0}},\frac{z-z_{n}}{d_{0}}\right)\text{, |}x|\leq \frac{D_{0}}{2},\text{ |}y|\leq \frac{D_{0}}{2},\text{ |}z|\leq \frac{D_{0}}{2}\\ with\;rect\left(x,y,z\right)=\left\{\begin{array}{cc} 1, & \text{|}x|<1/2,\text{ |y}|<1/2,\text{ |y}|<1/2\\ 0, & \text{otherwise} \end{array}\right) \end{array}$$

where * denotes the convolution, and V [x_n_, y_n_, z_n_] connotes the voxelization of V(x, y, z). It is shown that the voxelization operation (denoted by squared brackets "[ ]") suppresses the intravoxel details and produces a digital image representation of the vasculature configuration in the FOV. By applying voxelization to the 3D distributions of NAB(x, y, z), Δχ(x, y, z), ΔB(x, y, z), we obtain the corresponding 3D matrices of NAB[x_n_, y_n_, z_n_], Δχ[x_n_, y_n_, z_n_], and ΔB[x_n_, y_n_, z_n_], respectively. For example, in our simulation (see later), the fieldmap is originally represented as a large matrix in size of 2048 × 2048 × 2048 (with a fine grid resolution as used for the digital FOV representation), which can be reduced a smaller matrix in size of 32 × 32 × 32 by voxelization with voxel size of 64 × 64 × 64.

#### Voxel signal calculation by intravoxel dephasing integration

Exposed to an inhomogeneous fieldmap, a proton precesses with a phase angle Δ*ϕ*(*x, y, z, T_E_*) = *γ *· Δ*B*(*x, y, z*)·*T_E_*, where *γ *is the gyromagnetic ratio. It is noted that the phase angle is different from the field value by a constant factor *γ*⋅T_E _(Larmor law). Due to the finite dimension of a voxel (for example, d_0 _= 128 micron in our simulation), its voxel signal is formed by a vector sum of all spin packets (or isochromats) inside the voxel, called intravoxel dephasing integration [19]. For a d_0 _× d_0 _× d_0 _voxel at discrete position [*x_n_, y_n_, z_n_*], its BOLD signal is formed by

(7) $$S\left[x_{n},y_{n},z_{n};T_{E}\right]=\underset{|x-x_{n}|,|y-y_{n}|,|z-z_{n}|<d_{0}}{∭}e^{i\gamma \cdot \Delta B\left(x,y,z\right)\cdot T_{E}}dxdydz$$

where the echo time T_E _is explicitly retained to remind of the T_E _dependence of voxel signal (as will be demonstrated in our simulation later).

#### From voxel signal values to a multivoxel image

After calculating the voxel signals for all voxels in the FOV (with a specific T_E_), we assemble the voxel signal values into a 3D MR matrix according to the voxelization scheme in Eq. (6) by

(8) $$C\left[x_{n},y_{n},z_{n};T_{E}\right]=\left\{\begin{array}{cc} S\left[x_{n},y_{n},z_{n};T_{E}\right]\text{,} & \text{|}x-x_{n}|<d_{0},\text{ |}y-y_{n}|<d_{0},\text{ |}z-z_{n}|<d_{0}\\ void, & \text{otherwise} \end{array}\right)\;$$

which is complex-valued and explicitly T_E_-dependent. Since the MR magnitude image contrast is due to a spatial distribution of voxel signal decay, we are concerned with the magnitude loss. For the phase image, we are concerned with the phase angle accumulation during the period of T_E_. The magnitude loss map and phase accumulation map for a given T_E _are calculated by

(9) $$\{\begin{array}{l} A[x_{n},y_{n},z_{n};T_{E}]=1-|C[x_{n},y_{n},z_{n};T_{E}]|/|C[x_{n},y_{n},z_{n};T_{E}=0]|\\ P[x_{n},y_{n},z_{n};T_{E}]=∠C[x_{n},y_{n},z_{n};T_{E}]-∠C[x_{n},y_{n},z_{n};T_{E}=0] \end{array}$$

Where |C[x_n_, y_n_, z_n_; T_E _= 0]|denotes the non-decay initial magnitude image and ∠C[x_n_, y_n_, z_n_; T_E _= 0] the initial phase image. Henceforth, we will denote the MR magnitude and phase images as A[x_n_, y_n_, z_n_; T_E_] and P[x_n_, y_n_, z_n_; T_E_], respectively.

### Backward mappings

In the forward BOLD fMRI calculation, we obtain a pair of MR magnitude image and phase image by Eq. (9). Considering the NAB[x_n_, y_n_, z_n_] (the voxelized version of the NAB(x, y, z) in Eq. (6)) as the neuronal origin, the conventional neuroimaging effort consists in establishing a backward mapping from the MR magnitude images to the neuronal origin, as designated by "A ~ NAB" in Figure 2. Under a linear neuruovascular coupling model, we simplify the mapping between the vascular response (Δχ[x, y, z]) and the neuronal origin (NAB[x, y, z]) by a linear mapping in Eq. (4). On one hand, Δχ represents the vascular response to the neuronal origin under a neurovascular coupling process; on the other hand, it plays a vascular origin for the T2*MRI detection. In this paper, we will focus on the backward mapping from the MR magnitude image to its vascular origin as designated by "A~Δχ" in Figure 2.

It is known that the underlying source of BOLD fMRI is the susceptibility-expressed BOLD state, which is highly dependent upon the vasculature configuration in the FOV. To reduce the effect of vessel randomness on our numerical simulation, we propose to measure the similarity of the MR magnitude image A[x_n_, y_n_, z_n_] and the predefined BOLD susceptibility map Δχ[x_n_, y_n_, z_n_] by a spatial correlation coefficient as defined by

(10) $$corrA\left(T_{E}\right)=\frac{\text{cov}\left(A\left[:;T_{E}\right],\Delta \chi \left[:\right]\right)}{std\left(A\left[:;T_{E}\right]\right)std\left(\Delta \chi \left[:\right]\right)}$$

Where cov(x, y) denotes the covariance between vector x and y, std(x) the standard deviation, and ":" a nD-to-1D operator (as used by Matlab language) that reorders a high-dimensional array entries into one dimensional vector. The correlation coefficient defined in Eq. (10) gives rise to corrA ∈ [0, 1] with corrA = 1 for a perfect match (linear mapping) and corrA ≠1 for mismatch (nonlinear mapping). Since there is little decay for a short relaxation time (A[x_n_, y_n_, z_n_; T_E_]→0 for T_E _→ 0), a meaningful corrA(T_E_) should be evaluated at a relative long T_E _(T_E _> 0). However, a long T_E _will also introduce a diffusion smearing effect, which is not addressed herein. It is noted that corrA defined in Eq. (10) is a numerical measure of the backward mapping "A~Δχ" in Figure 2, which connotes the magnitude-vs-susceptibility correlation.

Likewise, we can measure the spatial correlation between the MR phase image P[x_n_, y_n_, z_n_; T_E_] and the fieldmap ΔB[x_n_, y_n_, z_n_] by

(11) $$corrP\left(T_{E}\right)=\frac{\text{cov}\left(P\left[:;T_{E}\right],\Delta B\left[:\right]\right)}{std\left(P\left[:;T_{E}\right]\right)std\left(\Delta B\left[:\right]\right)}$$

Likely, corrP is a numerical measure of the phase-vs-fieldmap correlation, a backward mapping designated by "P~ΔB" in Figure 2.

In summary, our computational BOLD fMRI model can be used for backward mappings: magnitude-vs-susceptibility correlation (corrA) and phase-vs-fieldmap correlation (corrP) by Eqs. (10) and (11), respectively. The goal of conventional neuroimaging and brain mapping is to render a backward mapping from MR magnitude image to neuronal origin, which consist of two steps: from the MR magnitude image to its vascular origin (as designated by "A~Δχ" and numerically measured by corrA) and then from the vascular response to its neuronal origin. In this paper, we are concerned with the mapping of "A~Δχ", with which we show the effect of MRI technology on the imaging performance of BOLD fMRI.

## Results

The main parameters and their settings for the simulations we performed are listed in table 1.

The overall numerical simulation scheme is described by a flowchart in Figure 2. It starts with defining a NAB in a cortical FOV (Eq. (1)) and filling the FOV with randomly generated vascular networks (Eq. (2)). For digital geometry depiction of small vessels, the FOV is finely gridded and represented in a large support matrix in size of 2048 × 2048 × 2048 (grid resolution = 1 micron). Then the magnetic susceptibility expression of a neurovascular-coupled BOLD state is analytically described by a linear spatiotemporal modulation formula in Eq. (4). At a snapshot time, we predefine a 3D susceptibility distribution based on the following parameter settings: a Gaussian-shaped NAB, a vasculature-laden FOV (under control of *bfrac*(t) = 2%) and Y(t) = 0.6. Let B_0 _= 3 T, we calculate the fieldmap from a 3D susceptibility perturbation in size of 2048 × 2048 × 2048 (assuming the same support matrix as FOV) by Eq. (5). By spatially partitioning the FOV into voxels, we calculate the voxel signals for a T_E _by using the intravoxel dephasing integration in Eq. (7). At last, we assemble the voxel signal values into a 3D image matrix by Eq. (8). By repeating the multivoxel BOLD fMRI simulations for a range of T_E _setting (T_E _= 0:2:60 ms with an increment of 2 ms), for the different voxel sizes (128 × 128 × 128, 64 × 64 × 64, 32 × 32 × 32 micron^3^), and for different vessel sizes (radii = 2 and 4 micron), we obtain a collection of MR magnitude images and phase images; from which we may observe the effect of MRI technology on the imaging performance of BOLD fMRI with respect to the echo time T_E_, the image resolution, and the vessel size. In particular, we are concerned with the magnitude-vs-susceptibility correlation (corrA in Eq. (10)) and the phase-vs-fieldmap correlation (corrP in Eq. (11)). In what follows, we present the simulation results via figures.

Figure 5 shows a 3D geometry for a NAB embedded in a cortical FOV, in which the cortical vasculature is simulated with a mixture of randomly populated cylinders (radii = {2,4,8}micron). In order to show the effect of different vessel sizes, we carry out the simulations for monosized cortical vasculatures (with radii = 2 and 4 micron separately, under the condition of *bfrac *= 2%). Using monosized vasculature allows us to address the vessel-size effect on the BOLD fMRI signals (the BOLD fMRI nonlinearity due to large vessels [35]).

With 1-micron digital grid resolution, we originally represent a FOV with large support matrix in size of 2048 × 2048 × 2048. After the fieldmap calculation, we partition the fieldmap into multivoxel image arrays for three voxel sizes: 16 × 16 × 16 matrix (voxel size: 128 × 128 × 128 micron^3^), 32 × 32 × 32 matrix (voxel size: 64 × 64 × 64 micron^3^), and 64 × 64 × 64 matrix (voxel size 32 × 32 × 32 micron^3^). With these three voxel sizes, we demonstrate the effect of image resolution on BOLD fMRI.

**Figure 6:**
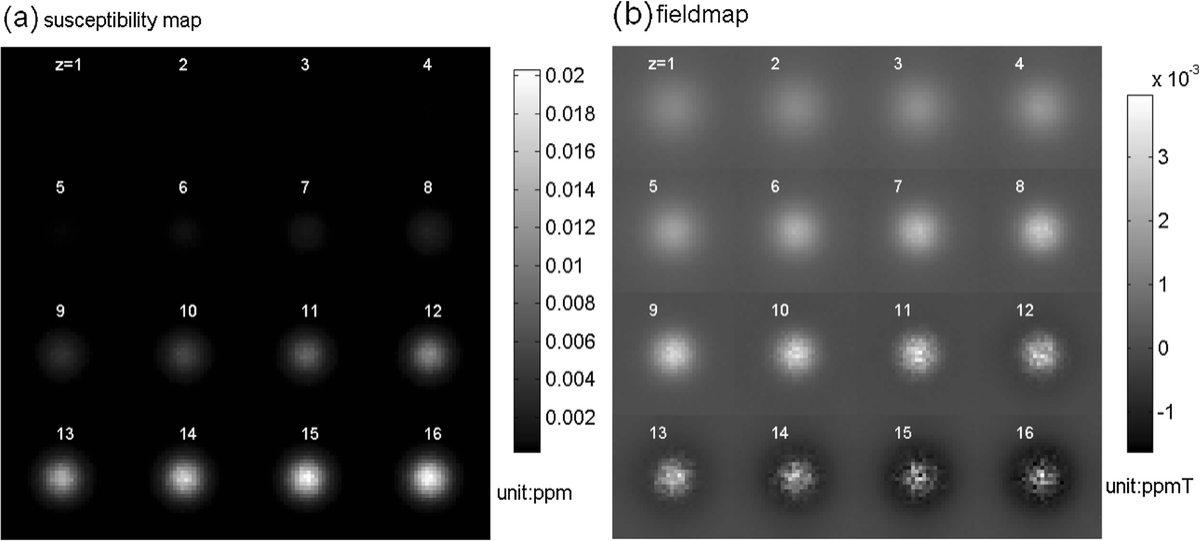
**Montage displays of (a) a 3D NAB-induced multivoxel susceptibility map Δχ (in size of 32 × 32 × 32 matrix) and (b) the corresponding multivoxel fieldmap ΔB (calculated for B_0 _= 3 T) for z-slices of z = [1,2,...,16] (z = 1 at FOV surface and z = 16 through FOV center)**. The 32 × 32 × 32 multivoxel matrix is calculated from a 2048 × 2048 × 2048 fine grid matrix (grid resolution = 1 micron), by voxelization with the voxel size of 64 × 64 × 64 micron^3^. The 3D NAB is a Gaussian-shaped blob embedded in the cortical FOV.

**Figure 7:**
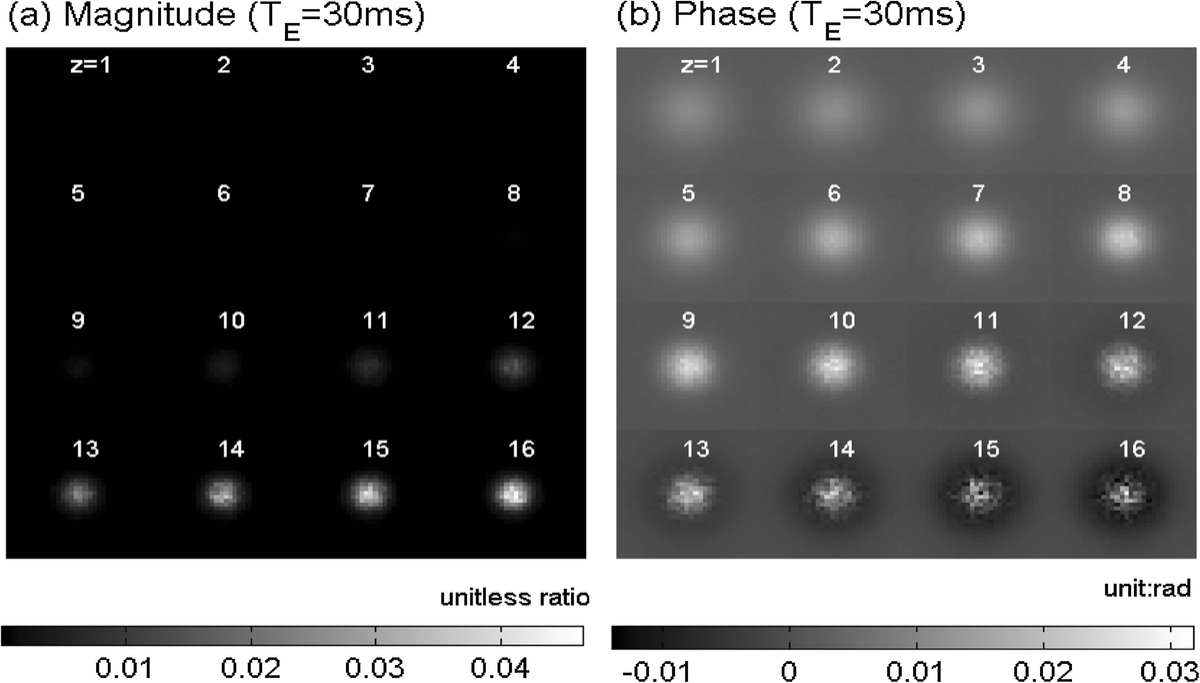
**Montage displays of (a) the 3D multivoxel magnitude image A[x, y, z] for z = 1:1:16 and (b) the multivoxel phase image P[x, y, z] (calculated with T_E _= 30 ms voxel size = 64 × 64 × 64 micron^3 ^and B_0 _= 3 T)**. Note that the central z-slice of the multivoxel image is at z = 16.

**Figure 8:**
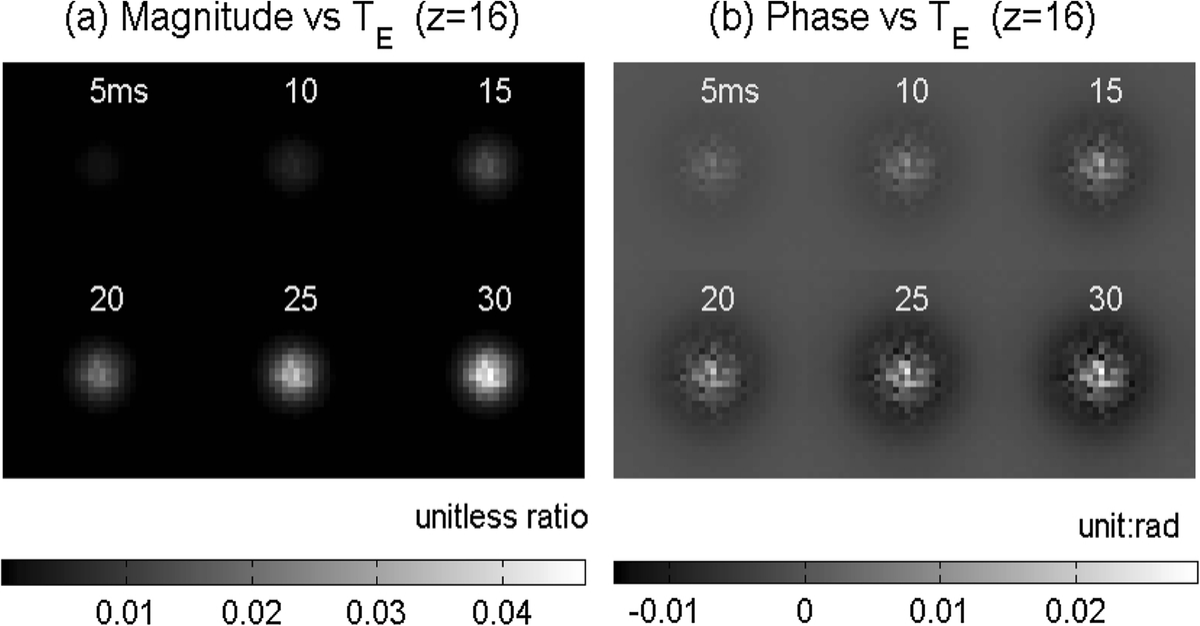
**Montage displays of the T_E _dependence of MR magnitude image at the central z-slice A[x, y, z_0_; T_E_] (in(a)) and the corresponding MR phase image P[x, y, z_0_; T_E_] (in (b)) for T_E _= **[5,10,15,20,25,30]**ms**.

Figure 6 shows the NAB-profiled susceptibility perturbation map (calculated by Eq. (4)) and the subsequent fieldmap (calculated by Eq. (5)). Figure 7 shows the MR magnitude and phase images (displayed with z-slices from FOV surface to the center surface) calculated at T_E _= 30 ms for voxel size = 64 × 64 × 64 micron^3^. It is noted that the magnitude image resembles the susceptibility map to a great extent, and the phase image replicates the fieldmap very well. Figure 8 shows the T_E _dependence of the MR magnitude and phase images (with the central z-slice).

**Figure 9:**
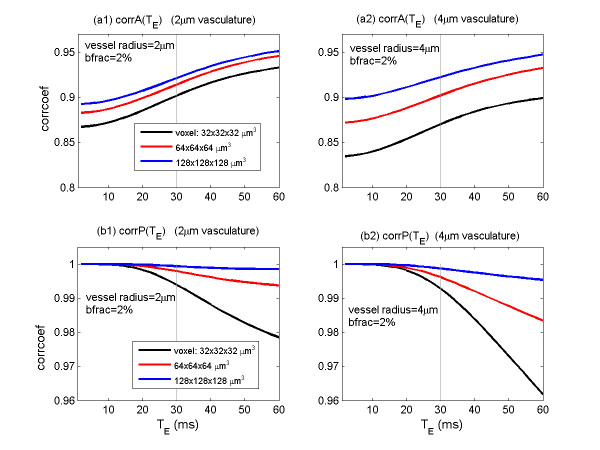
**(a1) and (a2) corrA for the magnitude-vs-susceptibility correlations with respect to T_E _= [0, 60] ms, two vasculatures of radii = 2 and 4 micron, and three voxel sizes (see legend); (b1) and (b2) corrP for the corresponding phase-vs-fieldmap correlations**. It is shown that a long T_E _may increase corrA but decrease corrP, and that both corrA and corrP decrease as image resolution refines. It is reminded that corrA≠1 implies mismatch and nonlinearity of the mapping.

With the MR magnitude and phase images generated for a variety of parameter settings, we calculate the magnitude-vs-susceptibility correlation and the phase-vs-fieldmap correlation by Eqs. (10) and (11), respectively. The results are shown in Figure 9 for T_E _in a range of 0 to 60 ms and three different image resolutions (see the legend). From Figure 9 we can see that the magnitude-vs-susceptibility correlation increases with respect to T_E _and voxel size; on contrary, the phase-vs-fieldmap correlation decreases with respect to T_E _and voxel size.

In Table 2 we present the correlation coefficients for two vasculatures (radii = 2 and 4 micron), three image resolutions (voxel size = 128 × 128 × 128, 64 × 64 × 64, and 32 × 32 × 32 micron^3^), and two selected echo times (T_E _= 1 ms and 30 ms).

**Table 2:** Spatial matching results in terms of spatial correlation coefficients corrA and corrP calculated by Eqs.(10) and (11) with the setting of B_0 _= 3 T, *bfrac *= 2%, radii = 2 and 4 μm, T_E _= 1 and 30 ms for three image resolutions (voxel sizes)

	2- μm-radius vessels				4- μm-radius vessels			
	**T_E _= 1 ms**		**T_E _= 30 ms**		**T_E _= 1 ms**		**T_E _= 30 ms**	

**Voxel size(μm^3^)**	**corrA**	**corrP**	**corrA**	**corrP**	**corrA**	**corrP**	**corrA**	**corrP**

32 × 32 × 32	0.867	1.000	0.885	0.998	0.834	1.000	0.854	0.998

64 × 64 × 64	0.883	1.000	0.899	0.999	0.871	1.000	0.887	0.999

128 × 128 × 128	0.892	1.000	0.907	0.999	0.898	1.000	0.910	0.999

## Discussion

In this paper, we propose a magnetic susceptibility expression formula for a linear neurovascular coupling model (accounting for the neurovascular aspects: CBF, CBV, CMRO_2_, FOV, and NAB) and a computational model for volumetric BOLD fMRI simulation. Overall, our goal is to numerically examine the principles of MRI-based neuroimaging: implementing the forward imaging from a neuronal origin (a numerical NAB), to vascular response (a numerical susceptibility distribution Δχ, which also serves as the vascular origin of T2*MRI), to complex multivoxel MR image formation (MR magnitude images), and then rendering the backward mappings (as designated by "A ~ NAB", "A~Δχ" and "P~ΔB" in Figure 2). In this paper, we focus on the mapping between the MR magnitude image and its vascular origin. Meanwhile, we also show the mapping between the MR phase image and the fieldmap.

We decompose the overall BOLD fMRI model into two modules in Figure 2: "neurophysiology" and "MRI technology". Due to the complexity of biophysiology and neurology, the neurovascular-coupled BOLD process is not completely understood. In contrast, the MRI technology is well understood and developed. We propose a linear neurovascular coupling formula to express a BOLD state in terms of susceptibility perturbation in Eq. (4), which accounts for the main aspects of BOLD process as parameterized by CBV, CBF, CMRO_2_, and NAB. It should be mentioned that the linear model neurovascular coupling model has the experimental supports [27,28,31,32]. For numerical simulation, we need to express the dynamics of neurovascular coupling process and BOLD fMRI through the digitization of the involved parameters at discrete time points (snapshots). It is straightforward to extend our snapshot simulation method to dynamic BOLD fMRI simulation by repeating the snapshot imaging at a series of time points.

The cortical vasculature mainly consists of capillaries with radii in a range from 2 to 10 micron. Voxelization may suppress the intravoxel vasculature details (due to voxel size > > vessel size), consequently, we cannot discern the vascular structures in Figures 1, 6 through 8. Our simulation results show that, the magnitude-vs-susceptibility correlation decreases for larger vessel sizes, which may in part explain the experimentally observed BOLD nonlinearity due to large vessel effect [35]. In general, the magnitude-vs-susceptibility correlation takes on a correlation coefficient of 0.90 for an image resolution about 100-micron. The discrepancy between the MR magnitude and the BOLD susceptibility map (corrA≠1) may be partially understood from the nonlinearity of BOLD fMRI [13,35]. We also demonstrate the phase-vs-fieldmap correlation. The results show that, the corrP is close to the maximum 1.00 with respect to T_E _(< 30 ms). However the corrP tends to drop for long T_E _and high resolution (see Figure 9(b) and Table 2 and refer to [20] for more relevant results and explanations).

In our simulation implementation, the cortex FOV is represented by a large 3D support matrix in size of 2048 × 2048 × 2048 (with 1-micron grid resolution) in order to depict small capillaries (digital geometry requirement). The output MR images are represented in much smaller multivoxel matrices (via voxelization): 16 × 16 × 16, 32 × 32 × 32, and 64 × 64 × 64 for three image resolutions. It is reminded that the fieldmap should be calculated from the finely-gridded susceptibility map (in the large support matrix) as a whole by a FT technique (in Eq. (5)), rather than trying to divide the large matrix into smaller submatrices. The 3D FFT on a large matrix (that is too large to be processed as a whole in computer memory) is implemented by a scheme of 2D + 1D decomposition [36]. By performing the 3D FFT on the large matrix, we can account for long-distance magnetic influence of susceptibility magnetization [22,23]. Indeed the multivoxel BOLD fMRI simulation demands a proliferated computation load in comparison with the single BOLD voxel signal simulation. Specifically, the volumetric simulation on an image matrix of 32 × 32 × 32 with voxel size of 64 × 64 × 64 (necessary for digital geometrical depiction of intravascular structure) requires a large matrix of 2048 × 2048 × 2048 (with 2048 = 64 × 32). Considering that the volumetric BOLD fMRI simulation may provide the spatial features among voxels that is not available from individual voxel signals, our simulation effort is worthwhile.

The simulation results for magnitude-based and phase-based backward mappings are graphically shown in Figure 9, from which we can observe the following phenomenon: 1) The magnitude-vs-susceptibility correlation (corrA) increases with respect to T_E _(shown in a range from 0 to 60 ms), whereas the phase-vs-fieldmap correlation (corrP) decreases for T_E _> 30 ms; 2) Large voxel size (or low image resolution) cause both corrA and corrP decrease (noticeable corrP decrease for T_E _> 30 ms); 3) Large vessels cause both corrA and corrP drop.

In addition to the study on the backward mapping between the MR magnitude image and its vascular origin, we also provide the backward mapping between the MR phase image and the fieldmap in a measure of corrP. Our simulation results show that corrP ≈ 1.00, which indicates that the MR phase image conforms very well with the fieldmap. This suggests the possibility of susceptibility reconstruction by computed inverse MRI [13,26], which is an active ongoing research topic.

Numerical simulation provides a powerful tool to look into a digital imaging system as long as all the involved parameters can be numerically characterized. In light of numerical method, a parameter must be digitized for computation. The parameter digitization does not necessarily require an analytic formula, it may be fulfilled in any way. For example, the BOLD susceptibility perturbation over a FOV in Figure 4 can be assigned a NAB-modulated spatial distribution at a snapshot time, and the dynamics can be numerically characterized by an empirical timecourse in a lookup table with respect to a discrete time (the voxel timecourses in Figure 4 are not necessarily analytically describable). Therefore, our computational model can be extended to accommodate a nonlinear neurovascular-coupled BOLD fMRI process over a broad physiological range provided that all the involved parameters can be numerically determined.

## Conclusions

In this report we propose a computational model for numerically simulating volumetric BOLD MRI with a magnetic susceptibility expression for a linear neurovascular coupling state. The forward procedure for this model includes: 1) defining a neuronal origin (a numerical NAB) in a cortex region(FOV); 2) expressing the NAB-modulated vascular response by a spatial distribution of intravascular blood biomagnetic susceptibility perturbation; and 3) calculating the susceptibility-induced fieldmap by accounting for the magnetization of all vascular blood in the FOV; 4) calculating the multivoxel MR image by intravoxel dephasing integration. Upon the completion of the forward simulation, we compare the output MR magnitude image with the predefined susceptibility map in a numerical measure of magnitude-vs-susceptibility correlation, thereby quantitatively examining the reproducibility of the vascular origin by the MR magnitude image. Meanwhile, we can also compare the output MR phase image with the precomputed fieldmap in terms of phase-vs-fieldmap correlation, showing that the MR phase image conforms with the fieldmap very well.

Based on our simulation results, we conclude that, 1) the magnitude image of BOLD fMRI can approximately, but not exactly, represent the vascular origin; and 2) the phase image conforms very well with the fieldmap. Considering that the vascular response to the neuronal origin is subject to a neurovascular coupling process, we can infer that the mapping between the MR magnitude image and neuronal origin suffers more mismatch than that between the MR magnitude image and the vascular origin, even if under a linear neurovascular coupling model (because the vasculature exerts a spatial modulation that imposes additional mismatch). The volumetric computational model provides a general framework for simulating many neurovascular, physiological, and biophysical aspects. It can be extended to accommodate a nonlinear neurovascular coupling process over a broad physiological range if the magnetic susceptibility expression is numerically available (not necessarily formulable). Our simulation results pose a caveat to the MRI-based neuroimaging and brain mapping study: the MR magnitude image is not an exact reproduction which may in part explain the nonlinearity of BOLD fMRI. In future research we plan to to look into the overall nonlinearity of BOLD fMRI by incorporating the intrinsic nonlinear neurovascular coupling process.

## Abbreviations

BOLD: Blood oxygenation level dependent; MR: Magnetic resonance; MRI: Magnetic resonance imaging; fMRI: Functional magnetic resonance imaging; FOV: Field of view; NAB: Neuroactive blob; CBV: Cerebral blood volume; CBF: Cerebral blood flow; CMRO_2_: Cerebral metabolic rate of oxygen; *bfrac*: Blood volume fraction.

## Competing interests

The authors declare that they have no competing interests.

## Authors' contributions

Zikuan Chen conceived the computational model, implemented the algorithm by program, and drafted the manuscript. Vince Calhoun analyzed the model, algorithm, data, and edited the manuscript. All authors have approved the content of the manuscript.

## Pre-publication history

The pre-publication history for this paper can be accessed here:

http://www.biomedcentral.com/1471-2342/12/8/prepub
